# Quantum Algorithm for Numerical Energy Gradient Calculations at the Full Configuration Interaction Level of Theory

**DOI:** 10.1021/acs.jpclett.2c02737

**Published:** 2022-11-29

**Authors:** Kenji Sugisaki, Hiroyuki Wakimoto, Kazuo Toyota, Kazunobu Sato, Daisuke Shiomi, Takeji Takui

**Affiliations:** † Department of Chemistry, Graduate School of Science, Osaka Metropolitan University, 3-3-138 Sugimoto, Sumiyoshi-ku, Osaka558-8585, Japan; ‡ JSTPRESTO, 4-1-8 Honcho, Kawaguchi, Saitama, 332-0012, Japan; § Centre for Quantum Engineering, Research and Education (CQuERE), TCG Centres for Research and Education in Science and Technology (TCG CREST), Sector V, Salt Lake, Kolkata700091, India; ∥ Research Support Department/University Research Administrator Center, University Administration Division, Osaka Metropolitan University, 3-3-138 Sugimoto, Sumiyoshi-ku, Osaka558-8585, Japan

## Abstract

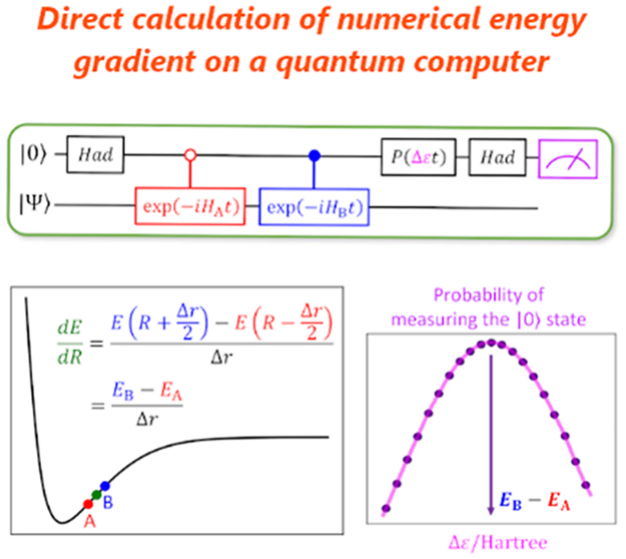

A Bayesian phase difference estimation (BPDE) algorithm allows us to compute the energy gap of two electronic states of a given Hamiltonian directly by utilizing the quantum superposition of their wave functions. Here we report an extension of the BPDE algorithm to the direct calculation of the energy difference of two molecular geometries. We apply the BPDE algorithm for the calculation of numerical energy gradients based on the two-point finite-difference method, enabling us to execute geometry optimization of one-dimensional molecules at the full-CI level on a quantum computer. Results of numerical quantum circuit simulations of the geometry optimization of the H_2_ molecule with the STO-3G and 6-31G basis sets, the LiH and BeH_2_ molecules at the full-CI/STO-3G level, and the N_2_ molecule at the CASCI(6e,6o)/6-311G* level are given.

Quantum computing is one of the most fascinating research fields in current science and technology.^1^ In particular, quantum chemical calculations of atoms and molecules are anticipated to be one of the most promising applications of quantum computers in the near future.^2−5^ Sophisticated quantum chemical calculations allow us not only to deeply understand chemistry and chemical phenomena from the first-principles point of view but also to design novel molecules and materials with exotic functionalities theoretically, bringing a paradigm shift in research and development in chemistry and related fields. The appearance of quantum hardware with high quantum volume^6^ and experimental demonstrations of quantum error corrections^7−9^ allow us to expect fault-tolerant quantum computing (FTQC) in the future. In this context, the development of quantum algorithms for the FTQC era is an urgent issue.

The full configuration interaction (full-CI) method can give the variationally best possible wave function within the basis set being used, and it is a practical goal of quantum chemical calculations. However, the computational cost of full-CI on classical computers scales exponentially with the number of basis functions relevant to the system size, and the method is impractical except for very small molecules. The situation has changed as a result of the appearance of a quantum phase estimation (QPE) algorithm^10^ that is capable of computing the eigenfunctions and corresponding eigenvalues of a unitary operator by utilizing measurement to project an approximate wave function onto the eigenfunction of the Hamiltonian. In 2005, Aspuru-Guzik and co-workers reported a method to perform full-CI calculations on a quantum computer using the QPE algorithm.^11^ Proof-of-principle experiments involving full-CI/STO-3G calculations on the H_2_ molecule using photonic^12^ and NMR^13^ quantum processors were reported in 2010. The QPE-based full-CI calculation requires too many quantum gates to handle on currently available noisy intermediate-scale quantum (NISQ) processors, but it is regarded as one of the most powerful approaches for quantum chemical calculations in the FTQC era. It should be noted that QPE is probabilistic and that the electronic state obtained depends on the square of the overlap between the approximate wave function used as the input and the true eigenfunction. The QPE itself does not guarantee an exponential speedup of quantum chemical calculations unless theoretical methods for sophisticated wave function preparation are established.

For the practical use of quantum chemical calculations, the development of geometry optimization methods^14^ is crucial because precise geometrical parameters are not always available from experiments. Geometry optimization is also important for the study of vibrational and thermodynamic properties and reaction discovery. Geometry optimization requires computation of the energy derivatives with respect to nuclear coordinates. Several approaches for energy derivative calculations based on the variational quantum eigensolver (VQE) have been reported,^15−24^ but in the VQE-based methods the measurement cost for energy expectation value evaluation can be a bottleneck when it is applied to systems with a large number of qubits.^25^ In QPE-based approaches, by contrast, the measurement cost is independent of the system size. In addition, due to its inherent projective nature, QPE is applicable with approximate wave functions, and variational full optimization of wave function is not necessary. However, analytical energy gradients are generally not available in QPE-based methods, and one has to rely on numerical derivatives. The putative approach based on the finite-difference method requires at least *d* + 1 evaluations of the energy, where *d* is the number of degrees of freedom. A pioneering work was reported in 2009 by Kassal and Aspuru-Guzik,^26^ who proposed a quantum algorithm that can compute the numerical energy gradient in a single query regardless of the dimension *d* of the system being investigated. However, in their quantum algorithm they assumed a black box (oracle) that computes the energy eigenvalue of an arbitrary input. The quantum algorithm developed in this study can be regarded as a special case of the black box for one-dimensional systems. In this paper we propose a theoretical approach to compute numerical energy gradients based on the two-point finite-difference method by utilizing the concept of a Bayesian phase difference estimation (BPDE) algorithm.^27,28^ The BPDE algorithm enables us to compute the energy gap of two electronic states of a given Hamiltonian without inspecting the total energies of the individual electronic states. In particular, here we extend the BPDE algorithm to the direct calculation of the energy gap of two different molecular geometries. It should be noted that theoretical methods for energy derivative calculations based on the Hellmann–Feynman theorem and eigenstate truncation approximation^15^ and finite-difference-based algorithms and the method based on the calculation of expectation values of force operators^16^ within the FTQC framework were discussed by O’Brien and co-workers.

Our quantum algorithm is an extension of the conventional Bayesian phase estimation (BPE) algorithm^29,30^ for total energy calculations as well as the BPDE algorithm for energy gap computations.^27^ The quantum circuits for the BPE-based full-CI calculations and the BPDE are illustrated in Figure 1a and Figure 1b, respectively. Detailed definitions of the quantum gates and quantum circuits are provided in the Supporting Information.

**Figure 1:**
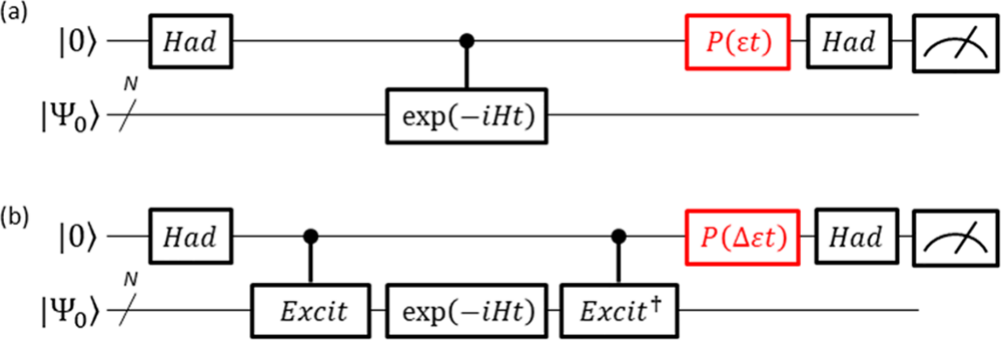
Quantum circuits for (a) the BPE-based full-CI calculations and (b) the BPDE-based full-CI energy gap computations. The parameter ε in the phase rotational gate shown in red is the estimator of the energy or energy difference, and it is optimized using Bayesian inference to maximize the probability to obtain the |0⟩ state in the measurement.

The BPE algorithm utilizes a controlled time evolution operation to extract the phase shift caused by the time evolution, which contains information on the eigenenergy. The eigenenergy readout is carried out by utilizing the phase rotation gate *P*(ε*t*) given in red in Figure 1a, where ε is a parameter used as the energy estimator. The quantum state before the measurement is given in eq 1:
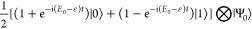
1The probability to obtain the |0⟩ state in the measurement of the top qubit in Figure 1a is calculated as in eq 2:
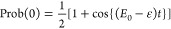
2From eq 2, Prob(0) becomes maximum when *E*_0_ = ε. In the BPE algorithm, the parameter ε is optimized to maximize Prob(0) using Bayesian inference. To do this, first we define a prior distribution Pr(ε) by a Gaussian function of which the mean μ corresponds to the initial estimate of the eigenenergy with a standard deviation σ. It should be noted that σ determines the width of the search area in Bayesian inference, and therefore, an initial value of the standard deviation must be large enough so that the actual value of *E* is located in the range between (μ – σ) and (μ + σ). After that, we repeatedly execute the quantum circuit in Figure 1a with fixed *t* and different ε in the range between (μ – σ) and (μ + σ) and generate an ε versus Prob(0) plot. Then the plot is fitted by a Gaussian function, which is used as a likelihood function Pr(0|ε; *t*). An updated posterior distribution Pr(ε|0; *t*) can be obtained by Bayesian inference using eq 3,
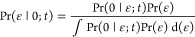
3and this posterior distribution is used as the prior distribution in the next step. This procedure is iterated until the standard deviation of the posterior distribution becomes smaller than the convergence threshold. More detailed procedures are given in the Supporting Information.

In the quantum circuit for the BPDE algorithm in Figure 1b, the quantum state in the superposition of |Ψ_0_⟩ and |Ψ_1_⟩ is generated using Hadamard (*Had*) and following controlled-*Excit* gates. Subsequently, quantum simulation of the time evolution is carried out unconditionally. Applying the controlled-*Excit*^†^ gate and following phase rotation *P*(Δ*εt*) and *Had* gates, the quantum state in eq 4 is obtained:
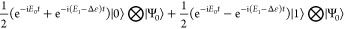
4where *E*_0_ and *E*_1_ are energy eigenvalues of |Ψ_0_⟩ and |Ψ_1_⟩, respectively. The probability to measure the |0⟩ state, Prob(0), is calculated as in eq 5:


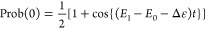
5Thus, we can compute the energy gap Δ*E* = *E*_1_ – *E*_0_ directly by finding the phase rotation angle Δε*t* that maximizes Prob(0) using the same procedure as in BPE, as described in detail in Supporting Information (SI) section 2.

In both the BPE and the BPDE algorithms, quantum simulation of the (controlled) time evolution of the wave function is involved. The following procedure is usually adopted to simulate the time evolution on a quantum computer: (i) The second-quantized electronic Hamiltonian shown in eq 6 is transformed to the qubit Hamiltonian in eq 7, which involves a linear combination of direct products of Pauli operators (Pauli strings), given in eq 8:
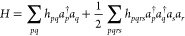
6
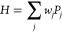
7
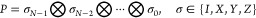
8where *N* is the number of qubits used for wave function storage and *I*, *X*, *Y*, and *Z* are the identity, Pauli *X*, Pauli *Y*, and Pauli *Z* operators, respectively. In this study, we used the Jordan–Wigner transformation^11,34^ for wave function mapping, and *N* is equal to the number of spin orbitals in the active space. (ii) The Trotter–Suzuki decomposition^31,32^ is then applied to the time evolution operator as shown in eq 9:
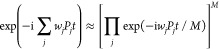
9(iii) Finally, the quantum circuit corresponding to the operator exp(−i*w*_*j*_*P*_*j*_*t*/*M*) is constructed.^33^ The quantum circuit for the time evolution operator corresponding to (controlled) exp(−i*wX*_0_*X*_1_*Y*_2_*Y*_3_*t*) is illustrated in Figure 2.

**Figure 2:**
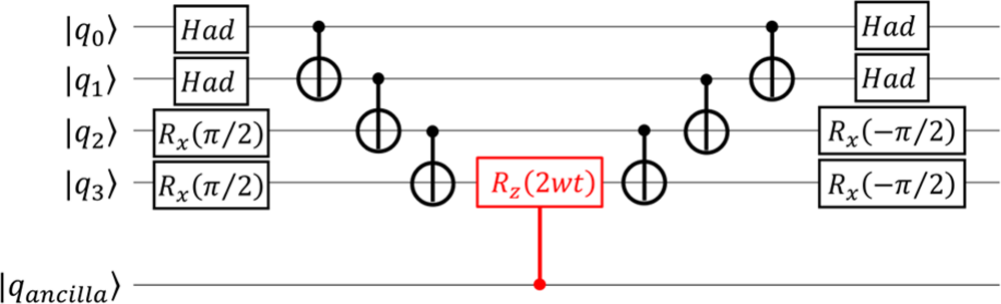
Quantum circuit corresponding to the (controlled) exp(−i*wX*_0_*X*_1_*Y*_2_*Y*_3_*t*) operation. *w* in the *R*_*z*_ gate is the coefficient of the Pauli string *X*_0_*X*_1_*Y*_2_*Y*_3_. The controlled *R*_*z*_ gate in red should be replaced by the *R*_*z*_ gate in the quantum circuit for the unconditional exp(−i*wX*_0_*X*_1_*Y*_2_*Y*_3_*t*) operation.

To calculate the energy difference of two geometries, we have to simulate the time evolution described in eq 10:
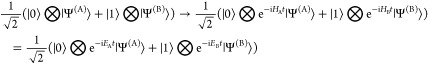
10where *H*_A_ and *H*_B_ are the Hamiltonians for the two geometries A and B, respectively, and |Ψ^(A)^⟩ and |Ψ^(B)^⟩ are the wave functions of the target electronic states at geometries A and B, respectively. |Ψ^(A)^⟩ and |Ψ^(B)^⟩ are generally different, but we can use the same |Ψ⟩ as the approximate |Ψ^(A)^⟩ and |Ψ^(B)^⟩ in the numerical energy gradient computation with the finite-difference method. Naive implementation of the operations in eq 10 involves applying two controlled time evolution operators, as illustrated in Figure 3a. Here the controlled time evolution gate with a closed (open) circle applies the time evolution operator to |Ψ⟩ if and only if the control qubit is in the |1⟩ (|0⟩) state. However, this implementation roughly doubles the depth of the quantum circuit, implying no advantage over the traditional two separate total energy calculations. We avoid this issue by invoking the following techniques.

**Figure 3:**
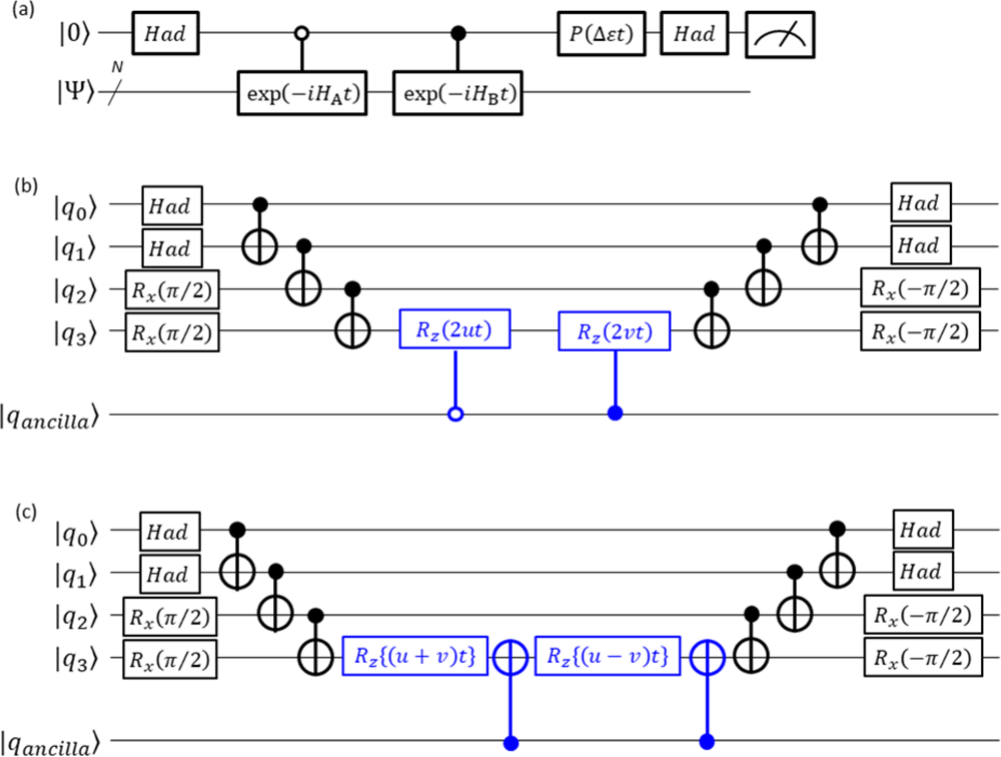
Quantum circuits used for the direct calculation of energy differences between two geometries. (a) Naive implementation. (b) Quantum circuit for the controlled time evolution operation (|0⟩⊗|Ψ⟩ + |1⟩⊗|Ψ⟩)√2 → (|0⟩⊗e^–i*uPt*^|Ψ⟩ + |1⟩⊗e^–i*vPt*^|Ψ⟩)√2 with *P* = *X*_0_*X*_1_*Y*_2_*Y*_3_ using two controlled *R*_*z*_ gates (see eq 13), where *u* and *v* in the *R*_*z*_ gates are the coefficients of the Pauli string *P*. (c) Another implementation of the quantum circuit for the controlled time evolution operation corresponding to eq 13 using two *R*_*z*_ gates and two CNOT gates.

We assume that the two Hamiltonians *H*_A_ and *H*_B_ share the same set of Pauli strings, as in eqs 11 and 12:
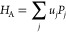
11
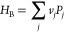
12The difference of the two Hamiltonians is fully characterized by the difference of the coefficients *u*_*j*_ and *v*_*j*_. As described above, these coefficients determine the rotational angle of the controlled *R*_*z*_ gate in the quantum circuit for the time evolution operations. Thus, the quantum circuit depicted in Figure 3a can be realized by applying controlled *R*_*z*_ operations with different rotational angles depending on the quantum state of the ancillary qubit. Figure 3b,c illustrates two possible implementations of the quantum circuit for the operation in eq 13:
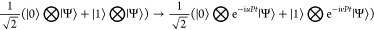
13where *P* = *X*_0_*X*_1_*Y*_2_*Y*_3_. Both of these implementations give the same quantum state. In these implementations, one controlled *R*_*z*_ gate in the quantum circuit for the time evolution operator (see Figure 2) is replaced by two controlled *R*_*z*_ gates (written in blue in Figure 3b) or two *R*_*z*_ gates and two CNOT gates (Figure 3c). Importantly, these implementations do not raise the scaling of the quantum gate count, and they slightly increase the proportionality factor of the gate count. As a result, the BPDE-based direct calculation of the energy difference of two geometries can be implemented with a computational cost slightly larger than that for the single-point-energy calculation. It should be noted that the quantum circuit described in Figure 3c has also been used in the implementation of model-state quantum imaginary time evolution (MSQITE).^35^

To demonstrate the quantum algorithm, we developed a Python program for numerical quantum circuit simulations using the PySCF,^36^ OpenFermion,^37^ and Cirq^38^ libraries. Detailed implementations of the quantum algorithm are given in the Supporting Information.

First we applied the BPDE-based numerical energy gradient calculation based on the two-point finite-difference method to H_2_ molecule at the full-CI/STO-3G level. The gradient is evaluated by using the central difference as in eq 14,
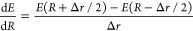
14in which the finite-difference value Δ*r* is equal to 0.0025 Å. The results of the numerical simulations are plotted in Figure 4, where the blue solid line specifies the energy gradient computed using the GAMESS-US program^39^ and the red circles represent the results of the quantum circuit simulations using the BPDE algorithm. The differences between the numerical energy gradient values computed using the BPDE algorithm and those calculated using GAMESS-US are shown in the inset of Figure 4. The BPDE algorithm can compute the numerical energy gradient to within an error of 0.02 hartree/Å. The BPDE-based calculations gave slightly large errors around *R*(H–H) = 1.0 Å. The standard deviations of the calculated d*E*/d*R* values for five runs were as small as 0.0006 hartree/Å for all points being investigated, and applying the finer Trotter decomposition did not improve the results (see SI section 4 for details). The deviation can be explained by the quality of the approximate wave functions used as the input. We used the two-configuration wave functions constructed using diradical character^40^ for the geometries *R*(H–H) ≥ 1.2 Å. The spin-restricted Hartree–Fock (RHF) wave functions were adopted for *R*(H–H) ≤ 1.1 Å because broken-symmetry UHF converges to the RHF solution for geometries with *R*(H–H) = 1.1 Å and shorter. It should be noted that the accuracy of the calculated gradient value is mainly controlled by the quality of the approximate wave function and that larger deviations were observed when we used the RHF wave functions for all geometries (see Figure S6). Adopting more sophisticated wave function preparation methods such as adiabatic state preparation (ASP)^11,13,41^ can further improve the accuracy. ASP-based wave function preparation may be necessary when the proposed method is applied to transition structure searches because transition states often have complicated electronic structures and the RHF approximation becomes worse.

**Figure 4:**
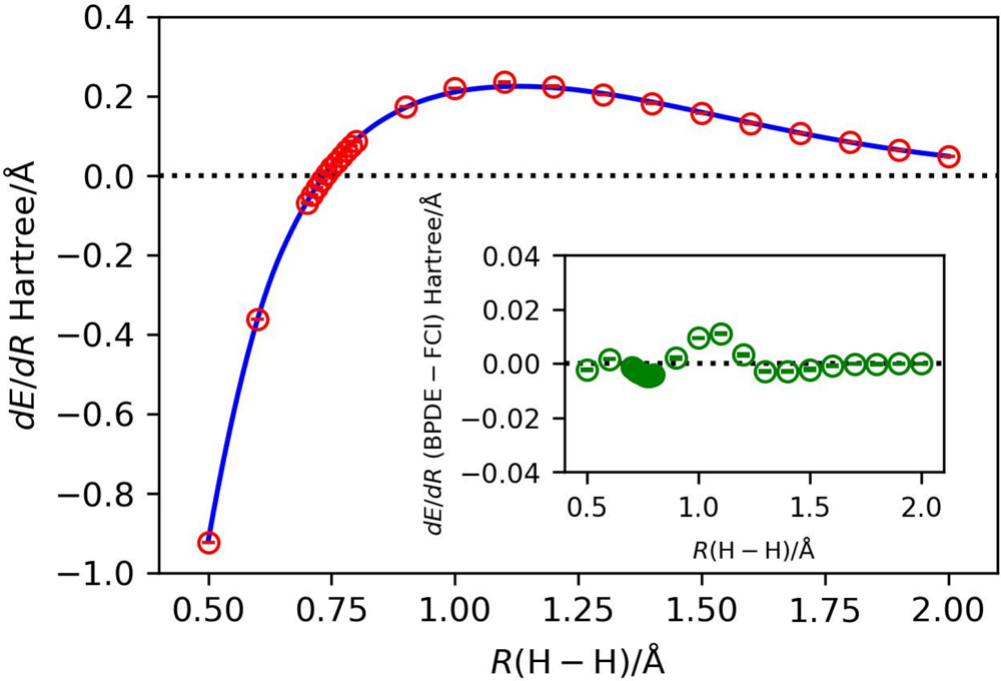
Numerical energy gradients of the full-CI/STO-3G potential curve for the H_2_ molecule. The blue line specifies the numerical gradient computed using the GAMESS-US program,^39^ and red circles represent the BPDE simulation results. The inset shows the differences between the gradient values computed from the BPDE numerical quantum circuit simulations and those calculated using GAMESS-US.

Because our BPDE-based method can compute numerical energy gradients accurately without inspecting total energies, we examined geometry optimizations of the H_2_ molecule using a gradient-only optimization algorithm that was developed by Wilke and co-workers to perform minimization of objective functions containing nonphysical jump discontinuities.^42^ Unlike the problems discussed by Wilke and co-workers, the full-CI potential energy curve for the H_2_ molecule does not contain discontinuities. However, the gradient-only optimization has an advantage of faster convergence compared with conventional gradient-based optimization because the gradient-only optimization adopts a three-point bisection interval, which is reduced by 50% after each iteration, as opposed to ∼38% for the golden-section search^43^ used in the gradient-based ones. It should also be noted that potential curve discontinuities can be present in VQE-based quantum chemical calculations with adaptive ansatzes^44^ when adaptive ansatz construction is adopted in each geometry.

Results of the numerical simulation of the geometry optimizations of the H_2_ molecule starting from different *R*(H–H) values are summarized in Figure 5, and SI section 5 provides additional details. Geometry optimizations were executed five times for each starting geometry. The BPDE-based geometry optimization converged after 5–10 iterations depending on the starting geometry, and the optimized value is *R*(H–H) = 0.736381 ± 0.000876 Å. The *R*(H–H) value optimized at the full-CI/STO-3G level using the GAMESS-US program is 0.734868 Å, and therefore, our BPDE gave the bond length with ca. 0.0015 Å error. It should be noted that in this study the threshold value of atom displacement was set to be 0.002 Å for the convergence check in the geometry optimization. It should also be noted that the BPDE-based numerical energy gradients with RHF wave functions tend to give a slight underestimate around the equilibrium geometry (see the Figure 4 inset), which is responsible for the deviation.

**Figure 5:**
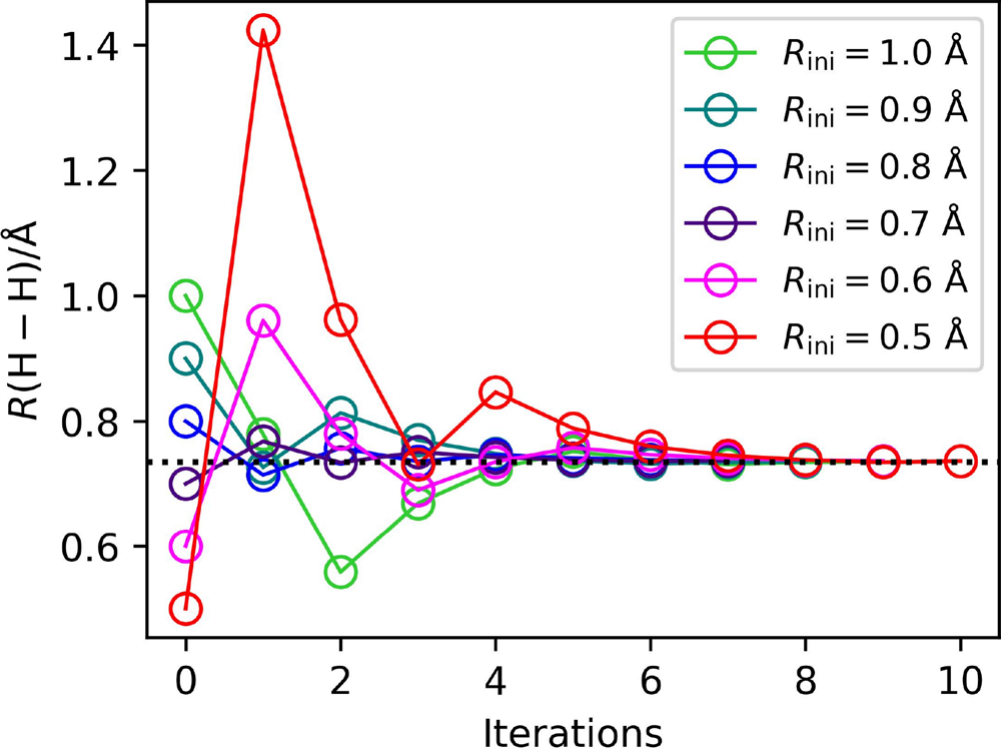
Results of the full-CI/STO-3G geometry optimizations using the BPDE-based numerical energy gradients. The black dotted line specifies the equilibrium bond distance calculated at the full-CI/STO-3G level using the GAMESS-US program.

To disclose the effect of the basis set, we tested the geometry optimization of the H_2_ molecule using the 6-31G basis set. The BPDE-based geometry optimization results in *R*(H–H) = 0.746242 ± 0.001274 Å, which is close to the full-CI/6-31G equilibrium bond length computed using the GAMESS-US software (0.746201 Å). We also applied the BPDE-based geometry optimization to the LiH and BeH_2_ molecules at the full-CI/STO-3G level and the N_2_ molecule at the CASCI(6e,6o)/6-311G* level. The optimized bond lengths are summarized in Table 1. The standard deviation of the equilibrium bond length of LiH is considerably large compared with the other molecules because of its rather shallow potential energy curve. Nevertheless, the geometry optimizations using the BPDE-based numerical energy gradients converged within ca. 0.015 Å deviations for all of the molecules studied.

**Table 1:** Optimized Bond Lengths of LiH, BeH_2_, and N_2_ Obtained from the Numerical Quantum Circuit Simulations of the BPDE Algorithm and Traditional Quantum Chemical Calculations

		*R*_eq_/Å	
molecule	computational level	BPDE	QC^a^
LiH	full-CI/STO-3G	1.561910 ± 0.002213	1.547516
BeH_2_	full-CI/STO-3G	1.323964 ± 0.000333	1.316476
N_2_	CASCI(6e,6o)/6-311G*	1.101648 ± 0.000729	1.107637^b^

a Calculated using the GAMESS-US program.

b The CASSCF(6e,6o)/6-311G*-optimized value.

Finally, we discuss possible implementation of the black box used in the quantum algorithm for the calculation of numerical energy gradients of *d*-dimensional systems by Kassal and Aspuru-Guzik.^26^ Their quantum algorithm starts with an equal superposition of *nd* qubits as in eq 15:
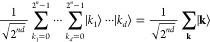
15where the states |*k*_*j*_⟩ are integers on *n* qubits represented in binary notation. The black box is used to compute the energy *E*(**μ**) at perturbation **μ** = *h*(**k** – **N**/2)/*N* in eq 16:
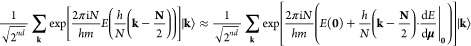
16where *h* is the finite-difference value and **N** denotes the vector (*N*, *N*, ..., *N*). From the analogy of the BPDE-based numerical energy gradient calculations, the black box can be implemented by substituting the controlled *R*_*z*_ gates in the quantum circuit for the time evolution by 2^*nd*^*R*_*z*_ gates conditional on *nd* control qubits. Such operations can be realized by 2^*nd*^ CNOT gates and 2^*nd*^ one-qubit rotation gates using the quantum circuit for general multiqubit gates discussed by Möttönen and co-workers^45^ based on the cosine–sine matrix decomposition technique.

In summary, we have developed a quantum algorithm for the direct calculation of the energy difference between two geometries and applied it to numerical energy gradient calculations based on the two-point finite-difference method and geometry optimizations of one-dimensional molecules. The scaling of the quantum gate count for the BPDE-based numerical energy gradient calculations is the same as that for single-point-energy calculations using the BPE algorithm. The geometry optimizations of the H_2_, LiH, BeH_2_, and N_2_ molecules converged within 10 iterations, and their optimized bond lengths match those obtained from traditional quantum chemical calculations within ca. 0.015 Å deviations. The proposed quantum algorithm is a special case of one-dimensional systems of the black box used in the quantum algorithm for numerical energy gradients proposed by Kassal and Aspuru-Guzik,^26^ and the possible construction of the black box for *d*-dimensional systems is discussed. Numerical energy gradients are used not only for equilibrium geometry optimizations but also for transition structure searches and molecular property calculations. Applications of the BPDE-based derivative calculations to these problems and the extension of the algorithm to higher-order derivatives such as Hessians are underway.

## Supporting Information Available

Definitions of quantum gates and quantum circuits, details of the implementation of the BPDE algorithm, computational conditions for numerical quantum circuit simulations, and results of the numerical energy gradient calculations and geometry optimizations of H_2_ molecule based on the BPDE algorithm (PDF)
