# Supplementary material for: Quantum Algorithm for Numerical Energy Gradient Calculations at the Full Configuration Interaction Level of Theory
Source: J Phys Chem Lett. 2022 Nov 29;13(48):11105–11. doi: 10.1021/acs.jpclett.2c02737 (PMC9743205; doi:10.1021/acs.jpclett.2c02737)
Supplement: Supplementary file 1 — jz2c02737_si_001.pdf [file jz2c02737_si_001.pdf]

Supporting Information

Quantum Algorithm for Numerical Energy Gradient Calculations at the Full Configuration Interaction Level of Theory

Kenji Sugisaki,[1,2,3*] Hiroyuki Wakimoto,[1] Kazuo Toyota,[1] Kazunobu Sato,[1*] Daisuke Shiomi,[1] and Takeji Takui[1,4*]

[1] *Department of Chemistry, Graduate School of Science, Osaka Metropolitan University, 3-3-138 Sugimoto, Sumiyoshi-ku, Osaka 558-8585, Japan*

[2] *JST PRESTO, 4-1-8 Honcho, Kawaguchi, Saitama, 332-0012, Japan*

[3] *Centre for Quantum Engineering, Research and Education (CQuERE), TCG Centres for Research and Education in Science and Technology (TCG CREST), Sector V, Salt Lake, Kolkata 700091, India*

[4] *Research Support Department/University Research Administrator Centre, University Administration Division, Osaka Metropolitan University, 3-3-138 Sugimoto, Sumiyoshi-ku, Osaka 558-8585, Japan*

* E-mail: sugisaki@omu.ac.jp; sato@omu.ac.jp; takui@omu.ac.jp

Table of Contents

		Page
1.	Definitions of quantum gates and quantum circuits	S2
2.	Implementation of the BPDE-based numerical energy gradient calculations	S4
3.	Computational conditions for the BPDE-based numerical energy gradient calculations and geometry optimizations	S7
4.	Results of the numerical energy gradients calculations based on the BPDE algorithm in H_2 molecule	S9
5.	Results of the geometry optimizations of H_2 molecule based on the BPDE-based numerical energy gradients and gradient-only optimization algorithm	S13
6.	References	S15

1. Definitions of quantum gates and quantum circuits

In quantum computers, qubits can be in an arbitrary superposition of the $|0\rangle$ and $|1\rangle$ states, as given in eq (S1).

$$|\varphi\rangle = c_0|0\rangle + c_1|1\rangle \quad \text{(S1)}$$

Here, c_0 and c_1 are arbitrary complex numbers satisfying a normalization condition given in eq (S2).

$$|c_0|^2 + |c_1|^2 = 1 \quad \text{(S2)}$$

The quantum state $|\varphi\rangle$ in eq (S1) can also be represented by a matrix as follows:

$$|\varphi\rangle = \begin{pmatrix} c_0 \\ c_1 \end{pmatrix} \quad \text{(S3)}$$

Quantum gates acting on one qubit can be expressed by a (2 × 2) unitary matrix and the quantum state after the quantum gate application can be calculated by matrix algebra. For example, the quantum state after the application of an Hadamard (*Had*) gate can be calculated as in eq (S4).

$$Had|\varphi\rangle = \frac{1}{\sqrt{2}}\begin{pmatrix} 1 & 1 \\ 1 & -1 \end{pmatrix}\begin{pmatrix} c_0 \\ c_1 \end{pmatrix} = \frac{1}{\sqrt{2}}\begin{pmatrix} c_0 + c_1 \\ c_0 - c_1 \end{pmatrix} \quad \text{(S4)}$$

The circuit symbols and matrix representations of the quantum gates frequently used for quantum chemical calculations are summarized in Table S1. As illustratively described in the controlled-R_z gates in Table S1, the conditional operation is executed if and only if the controlled qubit is in the $|1\rangle$ ($|0\rangle$) state when the circuit symbol for the controlled qubit is a close (open) circle. In the quantum circuit, the horizontal lines denote a qubit or *N*-qubits, and quantum gates are applied to the qubits from left to right order.

Table S1. Graph and matrix representations of quantum gates.

Gate	Circuit symbol	Matrix representation
Hadamard (*Had*)	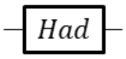	$\frac{1}{\sqrt{2}}\begin{pmatrix}1 & 1\\ 1 & -1\end{pmatrix}$
Pauli-*X*	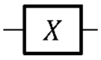	$\begin{pmatrix}0 & 1\\ 1 & 0\end{pmatrix}$
Pauli-*Y*	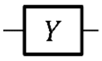	$\begin{pmatrix}0 & -i\\ i & 0\end{pmatrix}$
Pauli-*Z*	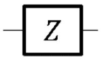	$\begin{pmatrix}1 & 0\\ 0 & -1\end{pmatrix}$
$R_x(\theta)$	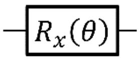	$\begin{pmatrix}\cos\frac{\theta}{2} & -i\sin\frac{\theta}{2}\\ -i\sin\frac{\theta}{2} & \cos\frac{\theta}{2}\end{pmatrix}$
$R_y(\theta)$	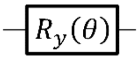	$\begin{pmatrix}\cos\frac{\theta}{2} & -\sin\frac{\theta}{2}\\ \sin\frac{\theta}{2} & \cos\frac{\theta}{2}\end{pmatrix}$
$R_z(\theta)$	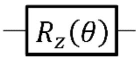	$\begin{pmatrix}e^{-i\theta/2} & 0\\ 0 & e^{i\theta/2}\end{pmatrix}$
Controlled-$R_z(\theta)$	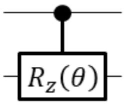	$\begin{pmatrix}1 & 0 & 0 & 0\\ 0 & 1 & 0 & 0\\ 0 & 0 & e^{-i\theta/2} & 0\\ 0 & 0 & 0 & e^{i\theta/2}\end{pmatrix}$
Controlled-$R_z(\theta)$	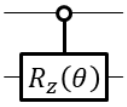	$\begin{pmatrix}e^{-i\theta/2} & 0 & 0 & 0\\ 0 & e^{i\theta/2} & 0 & 0\\ 0 & 0 & 1 & 0\\ 0 & 0 & 0 & 1\end{pmatrix}$
Phase shift $P(\theta)$	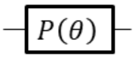	$\begin{pmatrix}1 & 0\\ 0 & e^{i\theta}\end{pmatrix}$
Controlled-NOT (CNOT)	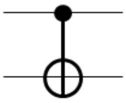	$\begin{pmatrix}1 & 0 & 0 & 0\\ 0 & 1 & 0 & 0\\ 0 & 0 & 0 & 1\\ 0 & 0 & 1 & 0\end{pmatrix}$

2. Implementation of the BPDE-based numerical energy gradient calculations

The BPDE-based numerical energy gradient calculation utilizes the quantum circuit given in Figure 3a in the main text, and two subsequent controlled-time evolution operations are implemented by using the quantum circuit depicted in Figure 3c. The wave function $|\Psi\rangle$ used as the input in the BPDE is described as the linear combination of eigenfunctions, as in eq (S5) and (S6) for geometry A and B, respectively. As discussed in the main text, we used $|\Psi^{(A)}\rangle = |\Psi^{(B)}\rangle = |\Psi\rangle$ for the finite difference-based numerical energy gradient calculations.

$$|\Psi^{(\mathrm{A})}\rangle = \sum_j c_j\, |\Psi_j^{(\mathrm{A})}\rangle \tag{S5}$$

$$|\Psi^{(\mathrm{B})}\rangle = \sum_k d_k\, |\Psi_k^{(\mathrm{B})}\rangle \tag{S6}$$

Here, $|\Psi_j^{(A)}\rangle$ and $|\Psi_k^{(B)}\rangle$ are the j-th eigenfunction at geometry A and the k-th eigenfunction of geometry B, respectively. Assuming eq (S5) and (S6), the probability of obtaining the $|0\rangle$ state in the measurement of the ancillary qubit in the quantum circuit in Figure 3a is calculated as in eq (S7).

$$Prob(0) = \left[1 + \sum_{j,k}\left|c_j\right|^2 |d_k|^2 \cos\left\{\left(E_k^{(\mathrm{B})} - E_j^{(\mathrm{A})} - \Delta\varepsilon\right) t\right\}\right] \tag{S7}$$

Here, $E_j^{(A)}$ and $E_k^{(B)}$ are the energy eigenvalues of the j-th electronic state at geometry A and the k-th eigenenergy at geometry B, respectively. From eq (S7), if the approximated wave function has sufficiently large overlap with the target electronic state at both geometries A and B, namely $|c_{\mathrm{target}}|^2 \sim |d_{\mathrm{target}}|^2 \sim 1$, the $Prob(0)$ becomes maximum around the point $E_{\mathrm{target}}^{(B)} - E_{\mathrm{target}}^{(A)} = \Delta\varepsilon$. From this, we can calculate the energy gap $E_{\mathrm{target}}^{(B)} - E_{\mathrm{target}}^{(A)}$ by optimizing the rotational angle of the phase shift gate $P(\Delta\varepsilon t)$ giving maximum $Prob(0)$, by means of Bayesian inference.

The BPDE-based numerical energy gradient calculation consists of the following steps. (1) Set the finite difference value Δr. (2) Define a prior distribution by a Gaussian function. (3) Set the evolution time length t from the standard deviation of the prior distribution σ. (4) Draw m samples in the range of $\mu - \sigma$ to $\mu + \sigma$ with a constant interval, and execute the quantum circuit R times with a given t and $\Delta\varepsilon$ to calculate a likelihood function $Pr(0|\Delta\varepsilon; t)$. Here, μ is a mean of the prior distribution. (5) Fit the obtained likelihood function by a Gaussian function and calculate a posterior distribution $Pr(\Delta\varepsilon|0; t)$. (6) If the standard deviation of the posterior distribution is smaller than the convergence threshold E_{Thre}, the algorithm returns the mean of the posterior distribution as the energy. Otherwise, the algorithm proceeds to the step (3) with the posterior distribution as the prior distribution of the next iteration.

In the calculations of numerical energy gradients with respect to nuclear coordinates, we adopted Δr = 0.0025 Å as the finite difference value in the step (1). This value is derived from preliminary numerical quantum circuit simulations (see Section 4 of this Supporting Information for details). Note that smaller Δr value can give the gradient value closer to the analytical one from the viewpoint of finite difference method, but the smaller Δr value gives the smaller ΔE value and therefore tighter

threshold for the convergence check E_{Thre} should be adopted to guarantee the quality of the derivative values. Because the computational cost of the BPDE-based energy gap calculation scales inversely proportional to the energy precision, there is a tradeoff between accuracy of the energy gradient value and the computational cost. It should be also noted that the similar values for atom position displacement are used in other quantum chemistry program packages (for example, 0.005 Bohr ≈ 0.00265 Å in ORCA software and 0.00374 Å for GAMESS-US program) for finite difference-based numerical gradients.

In the step (2), we set the initial mean of the prior distribution as 0 Hartree. The standard deviation of the prior distribution is defined as 1 Hartree. Note that the standard deviation determines the width of the search area in Bayesian inference, and therefore an initial value of the standard deviation must be large enough so that an actual value of ΔE locates in the range between $(\mu - \sigma)$ and $(\mu + \sigma)$. It should be also noted that the initial value of the standard deviation of the prior distribution is substantially large for the application of the gradient computation based on finite difference method, and smaller values such as 0.01 Hartree (≈ 6.27 kcal mol) may be enough.

In the step (3), the evolution time length t is set as in eq (S8). This condition is derived empirically so as to the likelihood function $Pr(0|\Delta\varepsilon; t)$ has a sufficiently large gradient and $Pr(0|\Delta\varepsilon; t)$ has a single maximum in the range of $\mu - \sigma \leq \varepsilon \leq \mu + \sigma$.

$$t = 1.8/\sigma \quad \text{(S8).}$$

In the step (4), we used m = 21 and R = 50000. We set the R value substantially larger than that used in the previous BPDE-based energy gap and full-CI energy calculations (R = 1000),[1,2] because we have to deal with very small energy differences in the finite difference-based gradient computations. Preliminary simulation results of the m and R dependences on the gradient values are given in Section 4 of this Supporting Information.

To execute the BPDE-based numerical energy gradient calculations, the molecular Hamiltonians H_A and H_B are transformed to the qubit Hamiltonian consisting of a linear combination of Pauli strings as in eq (11) and (12) in the main text, by using Jordan–Wigner transformation.[3] MO integrals appearing in the Hamiltonian were prepared by utilizing our own AO → MO integral transformation program, in conjunction with the one- and two-electron atomic orbital integrals computed by using GAMESS-US program package,[4] or computed by using PySCF program.[5]

The quantum circuit for the BPDE algorithm contains the controlled-time evolution operator U_A = $\exp(-iH_At)$ and $U_B = \exp(-iH_Bt)$. The quantum circuit corresponding to the controlled-U_A and controlled-U_B operators is constructed using the technique illustrated in Figure 3c in the main text, with the second-order Trotter decomposition given in eq (S9) with the time for a single Trotter step t/N = 0.5. It is known that Trotter decomposition error depends on the ordering of Trotterized terms to be applied.[6,7] In this work we used the magnitude ordering, in which Pauli strings are ordered by the absolute value of the sum of the norms of Pauli strings $|u_j| + |v_j|$.[7]

$$\exp(-i \textstyle\sum_m w_m P_m\, t) \approx \left[\prod_{m=1}^{M} \exp\left(-\frac{i w_m P_m t}{2N}\right) \times \prod_{m=M}^{1} \exp\left(-\frac{i w_m P_m t}{2N}\right)\right]^N \quad \text{(S9)}$$

In the step (5), a posterior distribution $Pr(\Delta\varepsilon|0;t)$ is calculated by using eq (S10).

$$Pr(\Delta\varepsilon|0;t) = \frac{Pr(0|\Delta\varepsilon;t)Pr(\Delta\varepsilon)}{\int Pr(0|\Delta\varepsilon;t)Pr(\Delta\varepsilon)d(\Delta\varepsilon)} \qquad \text{(S10)}$$

Because both the prior distribution $Pr(\Delta\varepsilon)$ and the likelihood function $Pr(0|\Delta\varepsilon;t)$ are given as Gaussian functions, we can easily calculate the posterior distribution.

The energy threshold used for the convergence check in the step (6) was set to be E_{Thre} = 0.0005 Hartree for all simulations. This threshold value is smaller than that used in the previous BPDE-based energy gap and total energy calculations, because we have to discuss small energy differences in the finite difference-based gradient calculations. The numerical quantum circuit simulation program was developed by utilizing OpenFermion[8] and Cirq[9] libraries in Python.

3. Computational conditions for the BPDE-based numerical energy gradient calculations and geometry optimizations

In this study the geometry optimizations of H_2, LiH, BeH_2, and N_2 molecules were performed using the numerical energy gradients computed from the BPDE quantum circuit simulations in conjunction with the gradient-only optimization algorithm. We employed the full-CI/STO-3G method in the geometry optimization of H_2, LiH, and BeH_2 molecules. The number of qubits used for wave function encoding is 4, 12, and 14 for H_2, LiH, and BeH_2, respectively. For H_2 molecule we also examined the geometry optimization at the full-CI/6-31G level using 8 qubits for wave function storage. In the geometry optimization of N_2 molecule we used the (6e,6o) active space consisting of valence σ/σ^* and π/π^* orbitals, in conjunction with the 6-311G(d) basis set. The active orbitals for the CASCI(6e,6o) calculations were selected from the RHF/6-311G(d) canonical orbitals.

Unless otherwise stated, the input wave functions of the BPDE-based numerical energy gradient computations were selected based on the following strategy. First, perform the broken-symmetry (BS) UHF calculation for the $M_S = 0$ state. If the BS-UHF calculation converges to the RHF solution, use the RHF wave function as the input of the BPDE calculations. In case the BS-UHF converges to the open shell states ($\langle \mathbf{S}^2 \rangle \neq 0$), construct the natural orbitals and calculate the diradical character y using eq (S11) in conjunction with the occupation number of the lowest unoccupied natural orbitals n_{LUNO}.[10] Then, the two-configurational wave function is constructed by using the quantum circuit depicted in Figure S1 and use it as the input wave function in the BPDE.

$$y = 1 - \frac{2(1-n_{\mathrm{LUNO}})}{1+(1-n_{\mathrm{LUNO}})^2} \quad \text{(S11)}$$

In the numerical energy gradient calculations of H_2 molecule with R(H–H) in the range from 0.5 Å to 2.0 Å those results plotted in Figure 4 in the main text and the geometry optimization of N_2 molecule at the CASCI(6e,6o)/6-311G(d) level of theory, the BS-UHF calculations were performed by using GAMESS-US program package. One- and two-electron MO integrals were prepared using in-house AO → MO integral transformation program, in conjunction with the one- and two-electron AO integrals computed by using GAMESS-US software. In the geometry optimizations of H_2, LiH, and BeH_2 molecules the RHF calculations were performed using PySCF program package, and corresponding one- and two-electron MO integrals were obtained from PySCF, by using OpenFermion-PySCF library. Because the likelihood function in the Bayesian optimization is calculated based on the finite number of sampling, the gradient values computed using the BPDE algorithm contain statistical errors. In the gradient calculations in Figure 4 in the main text, the numerical quantum circuit simulations were performed five times at each geometry. We confirmed that the standard deviations of five runs are less than 0.0006 Hartree/Å for all geometries being investigated.

In the geometry optimizations based on the numerical energy gradients computed from the BPDE algorithm, we used a gradient-only algorithm[11] for the molecular geometry update. In the gradient-only optimization algorithm the three-point bisection method is used instead of the golden-section

search to find an extremum. Detailed procedures for the gradient-only geometry optimization are as follows. (1) Calculate the gradient at the initial geometry. (2) Calculate the gradient at the geometry ($\boldsymbol{x} - l\boldsymbol{g}$), where $\boldsymbol{x}$ is the initial geometry, $\boldsymbol{g}$ is the gradient vector at the initial geometry. The line search iterations l are incremented until the sign of the gradient changes. (3) Refine the location of the sign change using the three-point bisection method until displacement of atoms becomes smaller than the threshold value R_{Thre}. In the present study we used R_{Thre} = 0.002 Å.

To investigate accuracy of the optimized geometries computed from the BPDE-based numerical energy gradients and gradient-only optimization algorithm, we also executed conventional geometry optimizations using GAMESS-US program package.

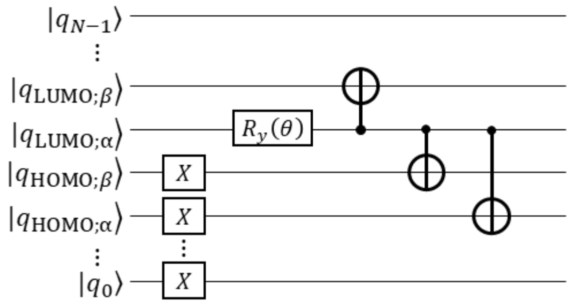

Figure S1. Quantum circuit used for preparation of the two-configurational wave function constructed by using diradical character y. The rotational angle θ of the $R_y(\theta)$ gate is set to be $\theta = -2\,\mathrm{acos}\left(\sqrt{1-y/2}\right)$. N is the number of qubits used for wave function storage. $|q_{\mathrm{HOMO};\alpha}\rangle$ represents the qubit storing the occupation number of α-spin orbital of HOMO, for example.

4. Results of the numerical energy gradients calculation based on the BPDE algorithm in H_2 molecule

In the BPDE-based numerical energy gradient calculations several computational conditions can affect the calculated derivative values. For example, the finite difference value Δr, the number of quantum circuit execution repetitions R, the number of sampling points m, the time length of single Trotter step t/N, and the wave function used as the input can influence the derivative values. In order to seek suitable computational conditions for numerical energy gradients, we performed several preliminary quantum circuit simulations in H_2 molecule.

First, the finite difference value dependence on the numerical energy gradient values was investigated by setting Δr = 0.0001, 0.0010, 0.0025, and 0.0050 Å. Other computational conditions were set to be m = 21, R = 50000, and t/N = 0.5. The deviations of the dE/dR values computed using the BPDE quantum circuit simulations from those calculated by using GAMESS-US program package are summarized in Figure S2. The simulations were executed five times for each geometry and averaged out, and the standard deviations of five runs were plotted. Figure S2 indicates that the smaller Δr value gave the larger errors of the dE/dR values. Especially, if Δr = 0.0001 Å is adopted the dE/dR values exhibit large standard deviations. This is because we used the same threshold value for the convergence check in the Bayesian optimization (E_{Thre} = 0.0005 Hartree) for all simulations, but this value is too small to predict the energy difference accurately when a small Δr value is used. We have to adopt tighter threshold values to calculate the gradient accurately for the smaller Δr values. Figure S2 indicates that Δr = 0.0025 or 0.0050 Å are suitable from both the viewpoints of mean values and standard deviations. We expect that Δr = 0.0050 Å may suffer from inaccuracy of the gradient values when strong anharmonicity is present on the potential energy surface. Thus, we adopted Δr = 0.0025 Å for the finite difference value for all simulations.

Results of the numerical simulation of the energy gradients of H_2 using difference number of quantum circuit repetitions are summarized in Figure S3. We used m = 21 and t/N = 0.5 for these simulations. As naturally expected, the smaller number of quantum circuit repetitions gave the larger standard deviations, although the mean of dE/dR values did not change so much.

Figure S4 summarizes the simulation results by changing the number of sampling points, while the total number of measurements is being approximately fixed (mR ~ constant). The time step for the single Trotter step was set to be t/N = 0.5. The results in Figure S4 imply that accuracy of the numerical energy gradient values are retained as long as the total number of measurements is the same.

To investigate the effect of Trotter decomposition errors, we performed numerical simulations with different t/N values (t/N = 0.1, 0.5, and 1.0). m and R were set to be 21 and 50000, respectively. The results are plotted in Figure S5. The numerical quantum circuit simulations with t/N = 1.0 gave considerably the larger errors compared with those with t/N = 0.1 and 0.5, especially in the region with the shorter H–H bond lengths. The simulation results with t/N = 0.1 and 0.5 almost coincide each other,

and therefore we concluded that the Trotter decomposition error is sufficiently small for t/N = 0.5 and shorter.

To check the input wave function dependence on the quality of the numerical energy gradient values in H_2 molecule, we performed the quantum circuit simulations using the RHF/STO-3G wave function as the input for all bond lengths. The results are summarized in Figure S6. Note that in Figure 5 in the main text, the two-configurational wave functions were used as the input wave functions in H_2 molecules with R(H–H) ≥ 1.2 Å. Clearly, the BPDE simulations with the RHF wave function gave large errors at the geometries R(H–H) ≥ 1.2 Å. Around this region the full-CI wave function cannot be well approximated by the single configuration, and HOMO–LUMO two-electron excited configuration contributes significantly to the full-CI wave function. As a result, the numerical gradient values computed by using the RHF wave function as the input in the BPDE contain non-negligible contributions from the second-excited singlet (S_2) state. The potential energy curve of the S_2 state is monotonically repulsive, and the dE/dR value is negative for all bond lengths. Thus, the dE/dR value computed by using the RHF wave function as the input shows a tendency of underestimation.

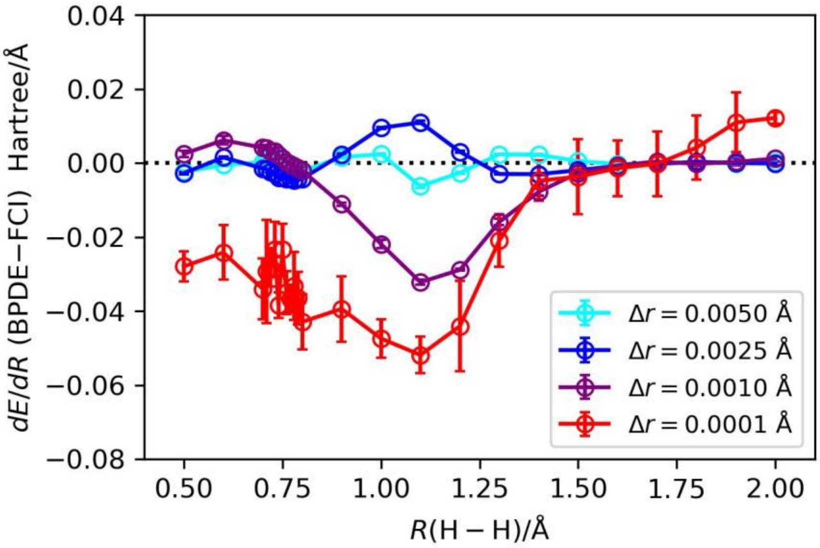

Figure S2. Deviations of the numerical energy gradient values of H_2 molecule at the full-CI/STO-3G level of theory computed by using the BPDE algorithm with different finite difference values from those calculated using GAMESS-US program package. Error bars specify the standard deviation of five runs.

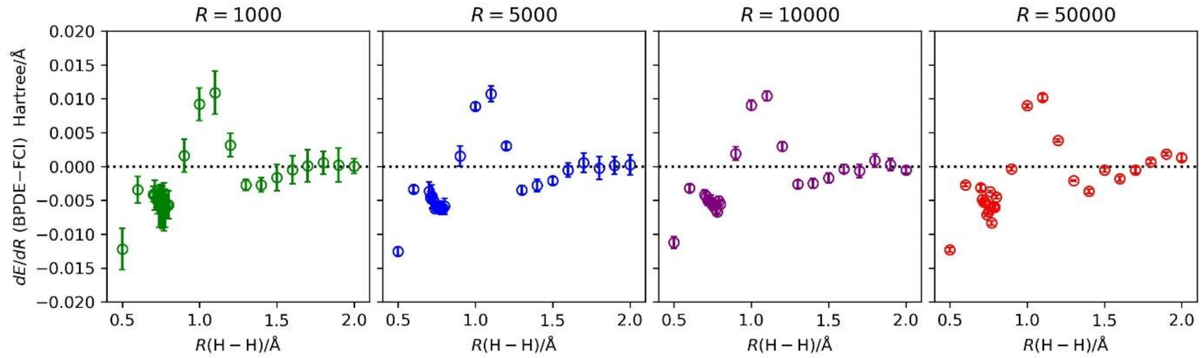

Figure S3. Results of the numerical quantum circuit simulation of the BPDE-based numerical energy gradient calculations of H_2 molecule with the different number of quantum circuit execution repetitions R. The number of sampling points m = 21 and time for the single Trotter step t/N = 0.5 were used. Error bars specify the standard deviation of five runs.

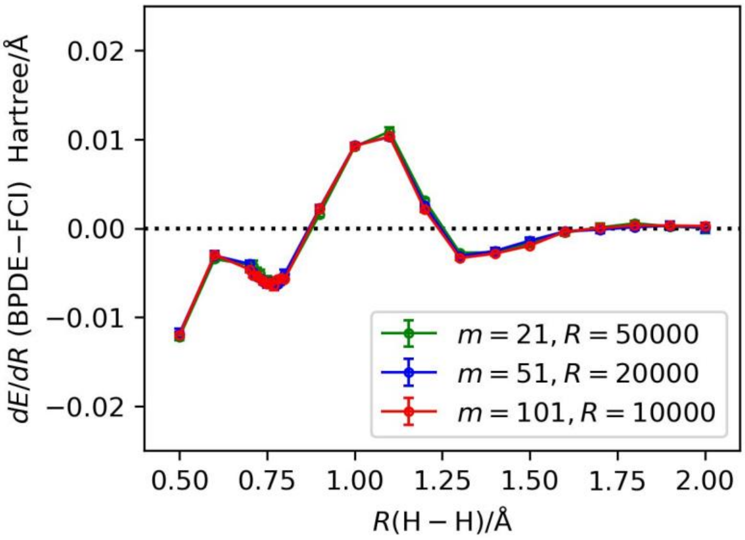

Figure S4. Results of the numerical quantum circuit simulation of the BPDE-based numerical energy gradient calculations of H_2 molecule with the different number of sampling points. The number of quantum circuit execution repetitions R is set so as to mR becomes almost constant. t/N = 0.5 is employed for all simulations. Error bars specify the standard deviation of five runs.

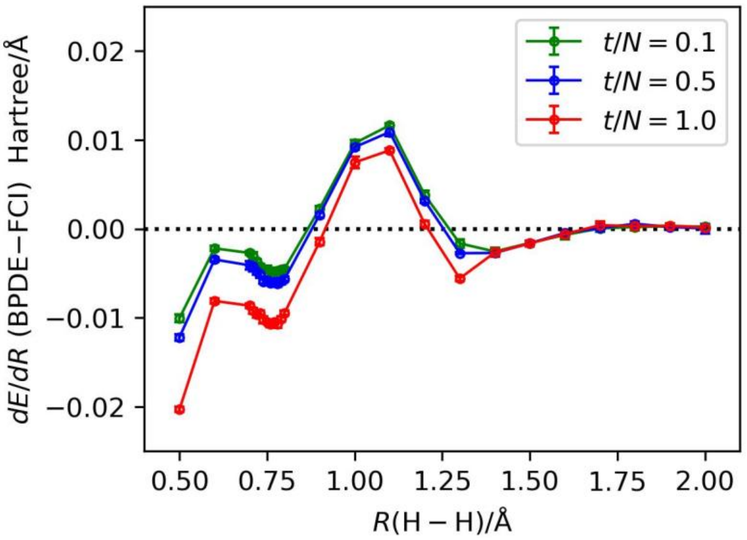

Figure S5. Results of the numerical quantum circuit simulation of the BPDE-based numerical energy gradient calculations of H_2 molecule with different time lengths for the single Trotter steps. R and m were set to be 50000 and 21, respectively. Error bars specify the standard deviation of five runs.

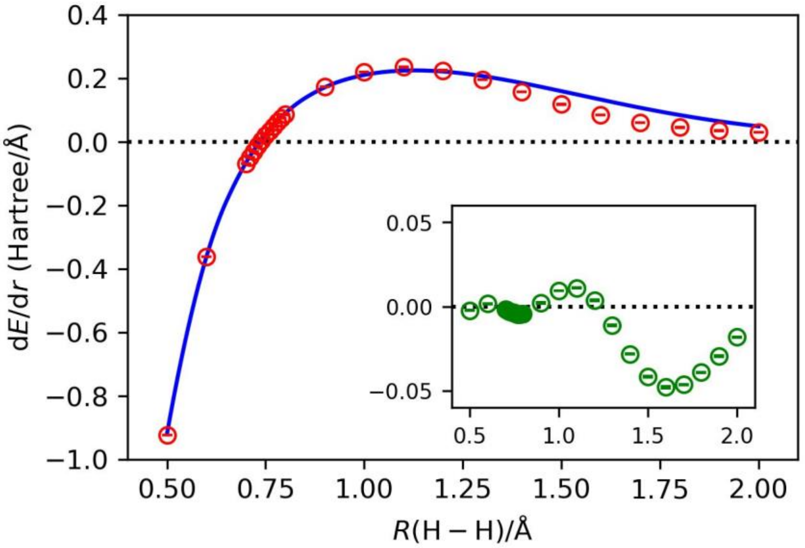

Figure S6. Results of the numerical quantum circuit simulation of the BPDE-based numerical energy gradient calculations of H_2 molecule with the RHF wave function as the input wave function. R, m, and t/N were set to be 50000, 21, and 0.5, respectively. Inset: The difference of the gradient values computed from two separate full-CI calculations and those from the BPDE numerical quantum circuit simulations.

5. Results of the geometry optimizations of H_2 molecule based on the BPDE-based numerical energy gradients and gradient-only optimization algorithm

In the geometry optimization of H_2 molecule at the full-CI/STO-3G level of theory, we examined six different initial geometries with R(H–H) = 0.5, 0.6, 0.7, 0.8, 0.9, and 1.0 Å, and geometry optimizations were performed five times for every geometry. The results of the geometry optimizations were plotted in Figure 5 in the main text, and the optimized bond lengths are summarized in Table S2. As discussed in the main text, the geometry optimization converged after 5–10 iterations, and the optimized bond length is calculated to be R(H–H) = 0.736381 ± 0.000876 Å. The optimized bond length at the full-CI/STO-3G level computed by using GAMESS-US is R(H–H) = 0.734868 Å. In order to disclose the origin of the difference of the optimized bond length between the BPDE-based quantum circuit simulations and traditional quantum chemical calculations, we have carried out geometry optimization using the gradient-only optimization algorithm in conjunction with numerical energy gradients computed from two separate full-CI/STO-3G calculations using GAMESS-US software. The same criteria for geometry optimization convergence were adopted. By setting the initial geometry as R(H–H) = 1.0 Å, the geometry optimization converged after 9 iterations, and the optimized bond length is R(H–H) = 0.734601 Å. Therefore, using the numerical energy gradient itself is not responsible for the deviation. As discussed in Figure 4 in the main text, the BPDE-based numerical energy gradient tends to negatively shift around the equilibrium geometry. As a result, the BPDE-based gradient predicts the equilibrium bond distance slightly longer than the numerical energy gradient computed from two separate full-CI calculations. As discussed in the previous section, increasing Trotter slices did not improve the gradient value so much, and therefore quality of the input wave function is majorly responsible for the deviation. We expect that using more sophisticated wave functions such as wave functions prepared by adopting adiabatic state preparation[12] as the input in BPDE will improve the gradient values and hence optimized geometries.

Table S2. Results of the geometry optimization of H_2 molecule at the full-CI/STO-3G level of theory in conjunction with the BPDE-based numerical energy gradient calculations and the gradient-only optimization algorithm.

Initial bond length/Å	Optimized bond length/Å	Number of geometry updates
0.5	0.736248	10
	0.736210	10
	0.736327	10
	0.732419	10
	0.736165	10
0.6	0.736516	9
	0.736313	9
	0.736449	9
	0.736510	9
	0.736500	9
0.7	0.737013	7
	0.737232	7
	0.737094	7
	0.737404	7
	0.737131	7
0.8	0.736931	7
	0.736351	7
	0.737157	7
	0.736607	7
	0.736737	7
0.9	0.735786	8
	0.735660	8
	0.736029	8
	0.737560	8
	0.735915	8
1.0	0.736168	9
	0.736370	9
	0.736498	9
	0.736133	9
	0.736001	9

6. References

(1) Sugisaki, K.; Sakai, C.; Toyota, K.; Sato, K.; Shiomi, D.; Takui, T. Bayesian phase difference estimation: a general quantum algorithm for the direct calculation of energy gaps. *Phys. Chem. Chem. Phys.* **2021**, *23*, 20152–20162.

(2) Sugisaki, K.; Sakai, C.; Toyota, K.; Sato, K.; Shiomi, D.; Takui, T. Quantum algorithm for full configuration interaction calculations without controlled time evolutions. *J. Phys. Chem. Lett.* **2021**, *12*, 11085–11089.

(3) Jordan, P.; Wigner, E. Über das Paulische äquivalenzverbot. *Z. Phys.* **1928**, *47*, 631–651.

(4) Barca, G. M. J.; Bertoni, C.; Carrington, L.; Datta, D.; De Silva, N.; Deustua, J. E.; Fedorov, D. G.; Gour, J. R.; Gunina, A. O.; Guidez, E.; Harville, T.; Irle, S.; Ivanic, J.; Kowalski, K.; Leang, S. S.; Li, H.; Li, W.; Lutz, J. J.; Magoulas, I.; Mato, J.; Mironov, V.; Nakata, H.; Pham, B. Q.; Piecuch, P.; Poole, D.; Pruitt, S. R.; Rendell, A. P.; Roskop, L. B.; Ruedenberg, K.; Sattasathuchana, T.; Schmidt, M. W.; Shen, J.; Slipchenko, L.; Sosonkina, M.; Sundriyal, V.; Tiwari, A.; Galvez Vallejo, J. L.; Westheimer, B.; Włoch, M.; Xu, P.; Zahariev, F.; Gordon, M. S. Recent developments in the general atomic and molecular electronic structure system. *J. Phys. Chem.* **2020**, *152*, 154102.

(5) Sun, Q.; Zhang, X.; Banerjee, S.; Bao, P.; Barbry, M.; Blunt, N. S.; Bogdanov, N. A.; Booth, G. H.; Chen, J.; Cui, Z.-H.; Eriksen, J. J.; Gao, Y.; Guo, S.; Hermann, J.; Hermes, M. R.; Koh, K.; Koval, P.; Lehtola, S.; Li, Z.; Liu, J.; Mardirossian, N.; McClain, J. D.; Motta, M.; Mussard, B.; Pham, H. Q.; Pulkin, A.; Purwanto, W.; Robinson, P. J.; Ronca, E.; Sayfutyarova, E. R.; Scheurer, M.; Schurkus, H. F.; Smith, J. E. T.; Sun, C.; Sun, S.-N.; Upadhyay, S.; Wagner, L. K.; Wang, X.; White, A.; Whitfield, J. D.; Williamson, M. J.; Wouters, S.; Yang, J.; Yu, J. M.; Zhu, T.; Berkelbach, T. C.; Sharma, S.; Sokolov, A. Y.; Chan, G. K.-L. Recent developments in the PySCF program package. *J. Phys. Chem.* **2020**, *153*, 024109.

(6) Tranter, A.; Love, P. J.; Mintert F.; Coveney, P. V. A comparison of the Bravyi–Kitaev and Jordan–Wigner transformations for the quantum simulation of quantum chemistry. *J. Chem. Theory Comput.* **2018**, *14*, 5617–5630.

(7) Tranter, A.; Love, P. J.; Mintert, F.; Wiebe N.; Coveney, P. V. Ordering of Trotterization: impact on errors in quantum simulation of electronic structure. *Entropy* **2019**, *21*, 1218.

(8) McClean, J. R.; Rubin, N. C.; Sung, K. J.; Kivlichan, I. D.; Bonet-Monroig, X.; Cao, Y.; Dai, C.; Fried, E. S.; Gidney, C.; Gimby, B.; Gokhale, P.; Häner, T.; Hadikar, T.; Havlíček, V.; Higgott, O.; Huang, C.; Izaac, J.; Jiang, Z.; Liu, X.; McArdle, S.; Neeley, M.; O'Brien, T.; O'Gorman, B.; Ozfidan, I.; Radin, M. D.; Romero, J.; Sawaya, N. P. D.; Senjean, B.; Setia, K.; Sim, S.; Steiger, D. S.; Steudtner, M.; Sun, Q.; Sun, W.; Wang, D.; Zhang F.; Babbush, R. OpenFermion: the electronic structure package for quantum computers, *Quantum Sci. Technol.* **2020**, *5*, 034014.

(9) Quantum AI team and collaborators. quantumlib/Cirq: Cirq, Version v1.0.0

(10) Yamaguchi, K. In *Self-consistent field: theory and applications*; Carbo, R., Klobukowski, M., Eds.; Elsevier: Amsterdam, 1990; pp 727−828.

(11) Wilke, D. N.; Kok, S.; Groenwold, A. A. The application of gradient-only optimization methods for problems discretized using non-constant methods. *Struct. Multidisc. Optim.* **2010**, *40*, 433–451.
(12) Sugisaki, K.; Toyota, K.; Sato, K.; Shiomi, D.; Takui, T. Adiabatic state preparation of correlated wave functions with nonlinear scheduling functions and broken-symmetry wave functions. *Commun. Chem*. **2022,** *5*, 84.
